# Estrogen Receptor-α in the Bed Nucleus of the Stria Terminalis Regulates Social Affiliation in Male Prairie Voles (*Microtus ochrogaster*)

**DOI:** 10.1371/journal.pone.0008931

**Published:** 2010-01-27

**Authors:** Kelly Lei, Bruce S. Cushing, Sergei Musatov, Sonoko Ogawa, Kristin M. Kramer

**Affiliations:** 1 Department of Biology, University of Memphis, Memphis, Tennessee, United States of America; 2 Department of Biology and Integrated Bioscience Program, The University of Akron, Akron, Ohio, United States of America; 3 Neurologix Inc, Laboratory of Neurobiology and Behavior, The Rockefeller University, New York, New York, United States of America; 4 Kansei, Behavioral and Brain Sciences Graduate School of Comprehensive Human Sciences, University of Tsukuba, Tsukuba, Ibaraki, Japan; Centre National de la Recherche Scientifique, France

## Abstract

Estrogen receptor alpha (ERα) typically masculinizes male behavior, while low levels of ERα in the medial amygdala (MeA) and the bed nucleus of the stria terminalis (BST) are associated with high levels of male prosocial behavior. In the males of the highly social prairie vole (*Microtus ochrogaster*), increasing ERα in the MeA inhibited the expression of spontaneous alloparental behavior and produced a preference for novel females. To test for the effects of increased ERα in the BST, a viral vector was used to enhance ERα expression in the BST of adult male prairie voles. Following treatment, adult males were tested for alloparental behavior with 1–3-day-old pups, and for heterosexual social preference and affiliation. Treatment did not affect alloparental behavior as 73% of ERα-BST males and 62.5% of control males were alloparental. Increasing ERα in the BST affected heterosexual affiliation, with ERα-BST males spending significantly less total time in side-by-side contact with females relative to time spent with control males. ERα-BST males did not show a preference for either the familiar or novel female. These findings differed significantly from those reported in ERα-MeA enhanced males, where ERα inhibited alloparental behavior and produced a preference for a novel female. The findings from this study suggest two things: first, that increased ERα in the BST decreases social affiliation and second, that altering ERα in different regions of the social neural circuit differentially impacts the expression of social behavior.

## Introduction

Estrogen acting via estrogen receptor alpha (ERα) masculinizes male behavior [1]-[4], which is typically associated with low levels of prosocial (positive affiliative) behavior and high levels of aggression. In male rats, treatment with a selective ERα agonist increased male aggression and anxiety [5] and masculinized serotonergic (5-HT) projections in female rats [6]. Conversely, data suggest that low levels of ERα are associated with high levels of male prosocial behavior. Highly social males, such as prairie voles (*Microtus ochragaster*), pine voles (*M. pinetorum*), and Djungarian hamsters (*Phodopus campbelli*), display low levels of ERα in two regions of the brain that play a critical role in the expression of social and sociosexual behavior, the bed nucleus of the stria terminalis (BST) and medial amygdala (MeA)[7]-[9]. In male prairie voles, increased ERα is associated with decreased prosocial behavior. Neonatal castration inhibits the expression of prosocial behavior in male prairie voles [10], [11], and significantly increases ERα expression in the BST and MeA [12]. Enhancing ERα in the MeA of male prairie voles decreased spontaneous alloparental behavior and resulted in a preference for a novel female over a familiar female [13]. These findings are significant for a couple of reasons. First, spontaneous alloparental behavior is extremely difficult to disrupt in male prairie voles [reviewed in 12]. Second, of the numerous studies testing partner preference in male prairie voles only one other study, in that case adrenalectomized males, has even reported a trend for a preference for the novel female [14].

Based on extensive overlap in function and interconnections between nuclei in the limbic system, the BST and MeA have been classified as part of a social neural network that also includes the medial preoptic area, lateral septum, ventromedial hypothalamus, and the anterior hypothalamus [15]. While social behavior requires the interplay of a number of regions of the brain, the BST and MeA may play a particularly critical role in regulating social interactions as these areas receive direct input from the accessory olfactory bulb, have bi-directional communication, and are among the first regions to show neuronal activation during social contact [16]–[19]. Not surprisingly, the BST and MeA have been implicated in regulating a variety of social and sociosexual behaviors, including social preferences, affiliation, aggression [15], [20], [21], and mating [22]; the MeA, in particular, is necessary for social recognition [21]. While many of these studies have been conducted in rats and mice, the MeA and BST are involved in regulating the same social behaviors in prairie voles [23]–[25], suggesting that these regions have a similar function in both highly social and less social species. Many of the studies that have examined the role of the BST in regulating social behavior have either found responses in both regions, which is not unexpected given the intimate relationships and interconnection, or have examined the response in only one region. One of the difficulties of interpreting the effects on behavior through the manipulation of a single region is that it is often unclear whether the change is due to the direct effect of the region or a response of the neural circuit to the manipulation. Therefore, one of the goals of this study is to use the same manipulation in a different region within the same neural circuit to determine if the effects are the same of if they vary by region.

Given that the BST and MeA have efferent and afferent neural connections and are part of the social neural network [15], ERα expression is low in both areas in male prairie voles [8], and ERα expression in the MeA decreased social behavior [13], we sought to determine the role of ERα in the BST. It also has been suggested that studying the role of ERα in the BST in regulating male social behavior is an essential next step [26]. Therefore the objectives of this study were to test the prediction that increasing ERα in the BST would reduce the expression of male prosocial behavior and to determine if the enhancing ERα in the BST had the same or different effects on male prosocial behavior as it did in the MeA. To accomplish this, a viral vector was used to enhance ERα expression in the BST of adult male prairie voles. This is a powerful technique for teasing apart the roles of specific brain regions as viral vectors can be delivered to specific sites using stereotaxic injection and they have limited spread, compared to the chronic and wide spread effect in knock out models. We replicated the design of Cushing et al. [13]. Treated males, along with the appropriate controls, were then tested for the expression of spontaneous alloparental behavior and heterosexual social preference and affiliation.

## Methods

### Subjects

Animals used in this study were laboratory-reared prairie voles that originated from wild stock trapped near Urbana, Illinois. Animals were housed under a 14∶10 light∶dark cycle and provided Purina high fiber rabbit chow (cat # 5326) and water *ad libitum*. Litters were weaned at 21 days of age and housed in same-sex pairs until treatment in polycarbonate cages (28.3 cm ×17.5 cm ×12.5 xm) with wood shavings for bedding. At the time of testing all subjects were sexually naïve adults, 60–90 days of age. Animals were maintained in accordance with USDA and NIH guidelines and all procedures were approved by the University of Memphis Institutional Animal Care and Use Committee prior to conducting any study.

### Viral Vector

Adeno-associated viral (AAV) vectors were used to enhance the expression of ERα in the BST. The following is a brief description of the vector; for complete details see Mustaov et al. [27]. The vectors express shRNA containing human ERα (AAV-ERα) or luciferase (AAV-luciferase) target sequences. Vectors also express enhanced green fluorescent protein (GFP) as a reporter that allows for visual detection of transduced neurons. These vectors have been previously demonstrated to permit effective transfection in prairie voles [13].

### ERα Adenoviral Vector Transfection

To achieve site-specific over-expression of ERα in the BST, adult males (60-70 days of age) were stereotactically injected, bilaterally, with AAV-ERα. A site-specific control was generated by transfecting the BST of males with a vector encoding firefly luciferase cDNA, and an ERα control was generated by transfecting the caudate putamen with ERα, as the caudate does not express ERα. At approximately 60 days of age, a stereotaxic apparatus was used for site-specific injections of the AAV vector into the brain of experimental males. Males were randomly assigned to one of three treatment groups: 1) AAV-ERα into the BST (n = 17), 2) AAV-ERα into the caudate putanum (ERα transfection control)(n = 9), or 3) AAV-luciferase into the BST (injection control)(n = 17). Prior to the procedure, animals were deeply anesthetized with a combination of Ketamine (67.7 mg/kg) and Xylazine (13.3 mg/kg). The viral vector (1 µl) was infused over a 5-min period with a micropump injector, and the infusion needle was left in place for an additional 3 min. The injection coordinates were determined to be AP −0.17 mm, ML±1.75 mm, 4.6 ventral from Bregma and, for the caudate, AP 1.3 mm anterior, ML±2.0 mm lateral, and 4.0 mm ventral. These coordinates specifically target the medial division of the BST. Animals were given 2–3 wks to recover and allow for expression of the vector prior to behavioral testing. Based upon a pilot study with prairie voles and other published results this amount of time is sufficient for the expression of transfected ERα and expression lasts at least13 weeks [27], [28]. Following completion of testing, animals were euthanized and their brains collected to verify the accuracy of the injection and successful transfection. Only animals with bilateral expression of transfected ERα were included in the analysis.

### Verification of Transfection

**Figure 1:**
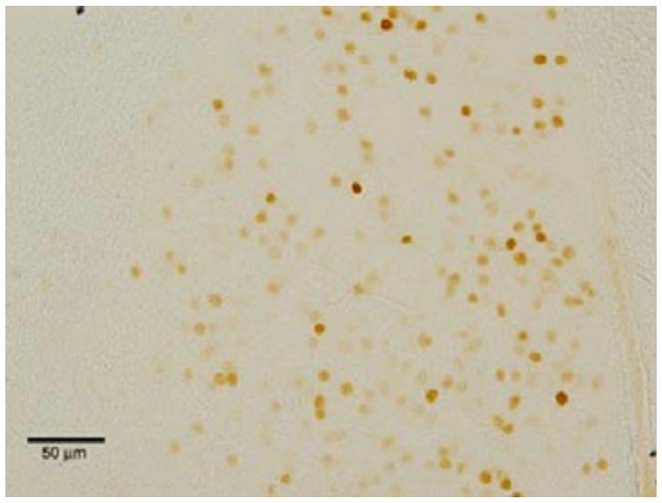
Shows photomicrograph of AAV-mediated ERα expression at 200x in the medial division of the BST. ERα was labeled using the human-specific antibody RM9101-s (Neomarkers, Fremont CA), which does not bind to endogenous prairie vole ERα. Immunoreactivity was visualized with DAB.

Upon completion of the social preference test, brains from experimental animals were fixed using immersion fixation, sectioned at 30 µm on a freezing-sliding microtome, and then the free-floating sections were stained for ERα using standard AB immunocytochemistry (for complete details see [8]). The human-specific primary antibody RM9101-s (Neomarkers, Fremont CA; 1∶1000 dilution) was used to label transfected ERα and then visualized using DAB. RM9101-s does not label prairie vole ERα. Therefore, all ERα observed with this antibody were the product of transfection. Successful transfection was determined qualitatively by visually examining ERα expression using a Nikon E-800 microscope. It should be noted that there were no animals that displayed only a few transfected-ERα immunoreactive cells; transfected males either displayed no or a significant amount of transfected-ERα cells. Fig. 1 shows a typical level of transfected ERα expression. Control BST transfection was determined by staining for GFP expression, as described in [13].

### Alloparental Behavior Test

Subjects were removed from the home cage and placed into a testing apparatus for 30–45 min to acclimate; food and water were provided during the acclimation period. The testing apparatus consisted of two standard size mouse cages (29 cm ×19 cm ×13 cm) connected by an 8-cm long clear acrylic tube. After the acclimation period, during which time all subjects investigated both mouse cages, an unrelated vole pup 1–3 days of age was introduced. The vole pup was placed in the cage without the subject so that latency to approach the pup could be assessed. Recording began as soon as the stimulus pup was introduced. The test continued for 10 min after the subject entered the cage with the pup. If an attack occurred, the test was ended immediately so that, if necessary, the pup could be treated and to prevent further injury. Subjects were given 30 min to make an approach before ending the test. All tests were recorded with a digital video camera and scored by the same experimentally-blind observer using the JWatcher program (UCLA). Behaviors scored and analyzed included latency to enter the pup cage, duration of pup-directed licking and huddling, and pup-directed attacks. Animals that displayed pup-directed aggression were classified as non-alloparental.

### Heterosexual Social Preference and Affiliation

One week after the alloparental test, treated males were tested for heterosexual social preference. This was done using the same apparatus as that used in the standardized vole partner preference test [11], [29]. Each subject was placed in a clean cage with a sexually naïve female for a 1-hr period of cohabitation. This female was designated as the “familiar.” Immediately after the cohabitation period, social preferences of the subject were assessed. The social preference arena consists of three polycarbonate standard size mouse cages in a modified Y-shape. The two cages housing stimulus animals are in parallel and a third cage (neutral) is attached separately to each stimulus cage by Plexiglas tubes. The familiar female is gently tethered in one of the stimulus cages while an age- and size-matched sexually naïve female, classified as “novel,” that is unrelated to both the familiar female and the subject is tethered in the other stimulus cage. At the start of the test, immediately after the cohabitation period, the experimental animal is placed in the neutral cage and is free to move among the three cages. The social preference test lasted 3 hrs and was recorded using a digital video camera and then scored using the JWatcher program at a 10∶1 temporal reduction. Behaviors scored included: time in each cage, time investigating each stimulus animal, and time spent in side-by-side contact with each stimulus animal. It should be noted that without hormonal manipulations, 1 hr of cohabitation is insufficient for prairie voles to form a preference for the familiar partner [14], [30] and so control males were expected to spend equal amounts of time with the familiar and novel females. All stimulus females were sexually naïve adults, 60–90 days of age, and mating is not a concern as female prairie voles do not undergo a spontaneous estrous cycle, requiring 24 or more hours of contact with a novel male before becoming sexually receptive [31].

### Statistical Analyses

For all data sets, the two control groups were compared using a *t*-test. In no case were there differences between the two control groups; these were combined into a single control group for all subsequent analyses. A Fisher's exact test was used to analyze whether or not there was a difference between the proportion of control and ERα-BST males that expressed alloparental behavior. A one-way ANOVA was used to compare alloparental behaviors, licking and huddling, by treatment. A one-way ANOVA was used to analyze between treatment effects on social preference, while a paired *t*-test was used to analyze within treatments effects. For all statistical tests, the criterion for significance was *P*≤0.05.

## Results

Eleven males were successfully transfected with ERα in the BST. Figure 1 shows a representation of successful transfection.

### Alloparental

**Figure 2:**
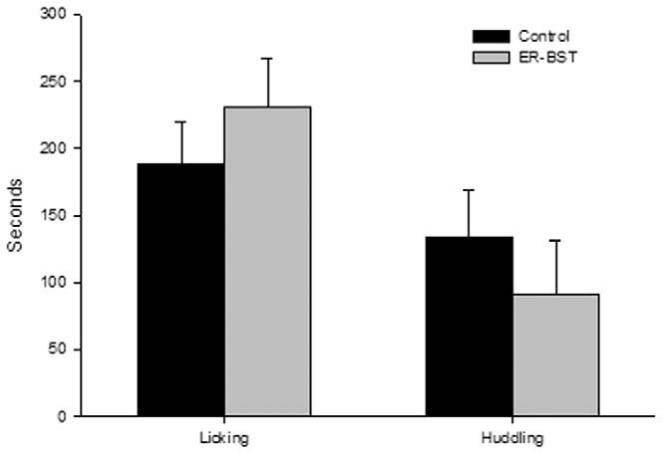
Show the mean (± s.e) time spent licking and huddling pups during the alloparental tests by treatment. There was no significant difference for either licking or huddling between control and ERα-BST males.

There was no significant difference in number of males that express alloparental behavior; 8 of 11 ERα-BST males were alloparental compared to 10 of 16 control males (Fisher's Exact *P* = 0.69). Non-alloparental behavior was comprised of, for ERα-BST males: 2 pup attacks and 1 with no contact; for controls: 2 pup attacks and 4 that did not enter the pup cage. In animals that expressed alloparental behavior, there was no effect of treatment on time spent licking or huddling with the pup (Fig. 2).

### Heterosexual Social Preference & Affiliation

**Figure 3:**
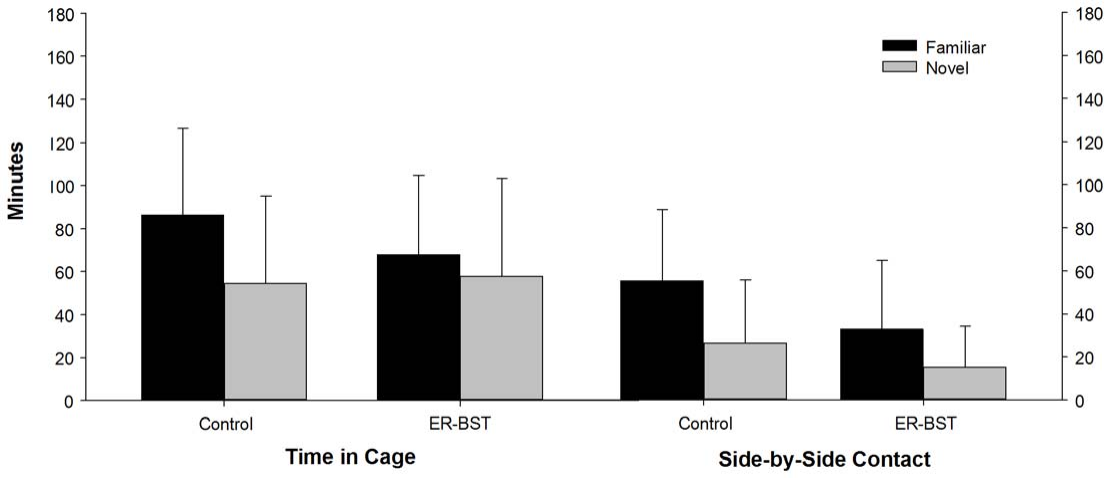
Shows the mean (± s.e) time spent in the cage of and in physical (side-by-side) contact with the familiar and novel stimulus female by treatment. While there was no significant difference either within or between treatments for time spent in the cage or in contact with the familiar versus the novel female ERα-BST treated males spent significantly less total time (combined familiar and novel) than control males (see text).

There was a significant effect of treatment on affiliation (Fig. 3). ERα-BST males (48±7.3 s.e. min) spent significantly less total time in side-by-side with both familiar and novel females than did control males (81.9±5.6 min; ANOVA *F*_1,25_ = 13.4 *P*<0.005; *t*_16_ = 3.43; *P* = 0.004). There was no significant difference between or within treatment groups for time spent in side-by-side contact with the familiar versus the novel female. Although a preference for the familiar animal is not expected after only 1 hr of cohabitation, control males did display a trend toward a preference for the familiar female (Control paired-*t*_16_ = −2.01; *P* = 0.06; ERα-BST paired *t*_9_ = −1.64; ns; Fig. 3).

## Discussion

The results from this study support the hypothesis that low levels of ERα in the BST play a role in the expression of male prosocial behavior. Increasing ERα in the BST significantly reduced heterosexual social affiliation, but did not impact the expression of alloparental behavior. These findings are significant on several levels. First, they suggest that mechanisms underlying different types of affiliation differ, as increasing ERα in the BST decreased physical contact with females, but did not alter the amount of time spent licking or huddling with pups. Second, the results differed significantly from the effects of enhancing ERα expression in the MeA, where enhanced ERα inhibited the expression of alloparental behavior and resulted in the formation of a preference of the novel female [13]. Although the BST and MeA are both part of the extended amygdala, these results indicate that they regulate different aspects of social behavior and that the MeA is more important in regulating the expression of allopaternal behavior than is the BST. Finally, the differential effects of increasing ERα in the BST versus in the MeA suggest the possibility that the suite of prosocial behaviors seen in male prairie voles is, at least in part, a product of the interaction between these two critical regions of the social neural circuit.

Enhancing ERα in the BST did not affect the expression of alloparental behavior. Male prairie voles typically display high levels of spontaneous alloparental behavior, ranging from 70 to 100% of males displaying alloparental behavior [13], [32]. While at the lower end of the range, 73% of ERα-BST males were alloparental. This is compared with 33% of males in which ERα was enhanced in the MeA [13]. The expression of male parental behavior has been associated with a number of regions in the brain, including the BST and the MeA. In prairie voles, male parental behavior was associated with increased fos expression in both the MeA and BST [16]. Also in male prairie voles, adult castration reduced the expression of male parental behavior and the expression of arginine vasopressin, which has been shown to play a significant role in male social behavior [33], [34], in the BST and MeA; treatment with testosterone restored both [23]. While Wang and De Vries [23] hypothesized that this resulted from the effect of testosterone on vasopressin expression, there is evidence that estrogen may play a direct role, with estrogen receptors increasing in the BST and MeA in male mice in response to interactions with pups [35]. While these studies did show changes in several regions of the brain associated with parental behavior, they did not differentiate the role of the individual regions. The findings from the current study, along with the previous examination of ERα manipulation in the MeA of male prairie voles, suggest that the MeA, and not the BST, is critical for the expression of paternal behavior. A significant role for the MeA in paternal behavior is supported by the finding that axon sparing lesions of the MeA disrupted male parental behavior [36]. It is, however, still possible that the BST plays a significant role in male parental behavior through the actions of other compounds such as arginine vasopressin [23], [37].

Enhancing ERα in the BST significantly reduced affiliation with females. This is in contrast to the effect of enhanced ERα in the MeA which did not affect total time spent in contact with females, but instead produced a preference for the novel female. Increased ERα expression resulted in a shift of affiliation, with male ERα-MeA males spending almost no time in contact with the familiar female [13]. In male prairie voles, neonatal castration not only inhibited the ability of central vasopressin to stimulate the formation of partner preferences [11], but also produced results similar to the current study, markedly reducing total side-by-side contact. Neonatal castration also resulted in a significant increase in ERα in both the MeA and the BST in adult males [12]. It is possible the results observed in the castration studies are the product of changes in both the BST and MeA, with increased ERα disrupting the formation of a partner preference, while changes in the BST reduced affiliation. This suggests that modification of prosocial behavior could result from subtle changes within one region of the brain, while changes in multiple regions my produce markedly different changes.

In conclusion, the ability to manipulate the expression of receptors in a site-specific manner can be a powerful tool. Studies that have looked at responses within the social neural circuit have been unable to differentiate the roles of the BST and the MeA. Although, it has been shown that there is a strong correlation between low levels of ERα expression levels in both the BST and MeA and the display of high levels of male prosocial levels ([7]–[9], our study indicates that the role of ERα is not redundant in these two brain areas. In terms of prosocial behavior a lack of ERα in the BST appears to facilitate heterosexual affiliation. Finally, future studies will examine the role of the BST in regulating male/male and heterosexual aggression. Reduced aggression is the other side of increasing prosocial behavior, and there are a number of studies that suggest that the BST may play an important role in regulating aggression [38]–[40].
